# Confirmed Autochthonous Case of Human Alveolar Echinococcosis, Italy, 2023

**DOI:** 10.3201/eid3002.231527

**Published:** 2024-02

**Authors:** Francesca Tamarozzi, Niccolò Ronzoni, Monica Degani, Eugenio Oliboni, Dennis Tappe, Beate Gruener, Federico Gobbi

**Affiliations:** IRCCS Sacro Cuore Don Calabria Hospital, Negrar di Valpolicella, Verona, Italy (F. Tamarozzi, N. Ronzoni, M. Degani, E. Oliboni, F. Gobbi);; Bernhard-Nocht-Institute, Hamburg, Germany (D. Tappe);; University Hospital of Ulm, Ulm, Germany (B. Gruener);; University of Brescia, Brescia, Italy (F. Gobbi)

**Keywords:** parasites, alveolar echinococcosis, *Echinococcus multilocularis*, Italy, autochthonous

## Abstract

In September 2023, a patient in Italy who had never traveled abroad was referred for testing for suspected hepatic cystic echinococcosis. Lesions were incompatible with cystic echinococcosis; instead, autochthonous alveolar echinococcosis was confirmed. Alveolar echinococcosis can be fatal, and awareness must be raised of the infection’s expanding distribution.

The main human echinococcal infections are caused by *Echinococcus granulosus* sensu lato, which causes cystic echinococcosis (CE), and *E. multilocularis*, which causes alveolar echinococcosis (AE). The parasites have different life cycles and cause different diseases (*1*). *E. granulosus* s.l./CE is endemic worldwide in livestock-raising areas, including Italy, and accounts for most human echinococcal infections (*2*). The parasite is transmitted in a domestic cycle between dogs and livestock and causes generally benign disease in humans marked by the formation of well-defined fluid-filled cysts mostly in the liver (*1*,*2*). *E. multilocularis*/AE is endemic to the Northern Hemisphere and is transmitted in a sylvatic cycle between wild canids (e.g., foxes) and small rodents (e.g., voles) (*2*).

Humans become infected with the 2 pathogens by accidental ingestion of parasite eggs from material contaminated with infected definitive host feces. In Europe, North America, and Asia, expanding distribution has been observed in recent decades (*2*). In Europe, the historical endemic areas are Austria, France, Germany, and Switzerland, and that range has expanded to include Eastern and Northern Europe (*3*). In Italy, infected foxes have been reported over the past 20 years in the Trentino-Alto Adige region (*4*–*7*). Autochthonous animal transmission might occur in the area (*8*), and prevalence in foxes seems to be increasing (*9*). A 2017 survey conducted in the Liguria region first detected *E*. *multilocularis* in fecal samples of dogs and wolves, suggesting a southward expansion of the parasite (*10*) (Appendix Figure), as predicted by modeling (*3*). Surveillance of *E. multilocularis* in Europe is usually conducted voluntarily (*11*), and no structured surveillance program for animal infection in Italy occurs beside targeted surveillance through research projects.

We report a confirmed autochthonous human AE case in Italy. Ethics approval was not necessary because data were derived from routine clinical practice. The patient consented to the publication of this report.

## The Study

In September 2023, a 55-year-old man in Italy was referred from his local hospital in Bolzano province, Trentino-Alto Adige region, to IRCCS Sacro Cuore Don Calabria Hospital, upon suspicion of CE. The patient was born and lived in Trentino-Alto Adige and had never traveled abroad; he worked in the tertiary sector and did not report contact with wild carnivores. He also did not report risk factors for *E. granulosus* infection.

The suspicion of CE was posed in June 2023, when he underwent abdominal ultrasound for a mild thrombocytopenia, revealing 5 recently developed small hepatic lesions (ultrasound results in 2016 were unremarkable). The lesions were described as septated and hypodense with no contrast enhancement and no calcifications on computed tomography performed in June 2023 (Figure, panels A–D), hypointense in T1-weighted magnetic resonance imaging (MRI), and hyperintense in T2-weighted MRI (Figure, panels E–H), with no diffusion restriction. No other lesions were present on total body imaging. Results of *Echinococcus* serologic testing using Western blot method were positive, but banding pattern was not reported.

**Figure:**
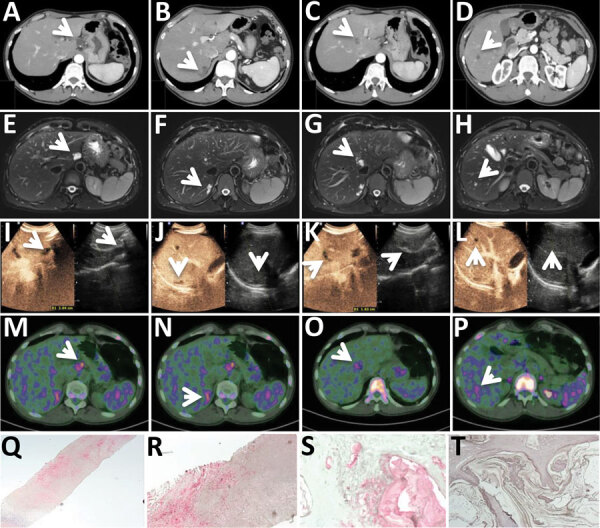
Diagnostic tests for patient in Italy with confirmed autochthonous case of human alveolar echinococcosis, 2023; white arrows indicate lesions. A–D) Contrast-enhanced computed tomography arterial phase. E–H) T2-weighted magnetic resonance imaging. I–L) Ultrasonography and contrast-enhanced ultrasonography. M–P) ^18^F-FDG-PET scan delayed acquisition (4 hours). Q–T) Em2 immunohistochemistry indicating small particles of *Echinococcus multilocularis* (spems) stained in red in patient’s sample (original magnification ×2.5 [Q] and ×20 [R]); positive alveolar echinococcosis sample control (original magnification ×20 [S]); Em2 negative control (cystic echinococcosis case, negative laminated layer; original magnification ×20) (T).

We excluded the diagnosis of CE on the basis of the lesions’ morphology on ultrasound, which did not show any CE pathognomonic or compatible features. We observed 5 lesions: 1 with 2.3 cm diameter in hepatic segment I, 1 of 0.9 cm in VI, 2 subcapsular of 2.7 cm and 0.5 cm in VII, and 1 of 1.6 cm in segment VIII (adjacent to the median hepatic vein). The lesions were hypoechoic with irregular margins, particularly the lesion in segment I, which had fine and tightly packed septations (Figure, panels I–L). The lesions were not enhancing on contrast-enhanced ultrasound (Figure, panels I–L). Serologic testing using the *Echinococcus* Western Blot IgG (LDBIO Diagnostics, https://ldbiodiagnostics.com) was positive for *Echinococcus* spp., showing genus-specific 7 kDa and 26–28 kDa bands, not assignable specifically to a species. Results of an ^18^F-FDG-PET scan showed light hypermetabolism in delayed (4-hour) acquisitions (Figure, panels M–P). Taken together, those results made the lesions highly indicative of AE. 

We performed a biopsy of the only accessible lesion, located in segment VI, and submitted the specimen to the German Reference Laboratory for Tropical Parasites at the Bernhard-Nocht Institute for Tropical Medicine (Hamburg, Germany). A serum sample also submitted for further serologic testing showed low antibody titers against crude antigen preparations of *E. multilocularis* and *E. granulosus* (1:40–1:80 in indirect hemagglutination [negative <1:20] and 30–40 arbitrary units in ELISA [negative <20]). Results of Em18-ELISA (*12*) were negative. Histology revealed necrotic granuloma and fibrosis without PAS-positive structures. Results of PCR testing targeting cestode cytochrome oxidase and *Echinococcus*-specific 12S rDNA (*13*) were negative. In contrast, immunohistochemistry with the monoclonal antibody Em2G11 (*14*) stained positive for small particles of *E. multilocularis* (spems) (Figure, panels Q–R). Spems consist of outer laminated layer of Em2 antigen in close proximity to AE lesions and thus confirmed the diagnosis of AE, defined by the WHO Informal Working Group on Echinococcosis (WHO-IWGE) as the presence of clinical-epidemiologic factors plus compatible imaging findings plus seropositivity for echinococcosis plus compatible histopathology (*15*). The laboratory uses Em2G11-IHC regularly for suspected AE. The immunohistochemistry has been extensively validated and is also used by other laboratories; CE lesions and other cestode lesions stain negatively (*14*), whereas lesions by neotropical *E. vogeli* stain faintly (Appendix reference *16*).

Staging according to the WHO-IWGE (*1*,*15*) was P2N0M0 (i.e., central lesions with proximal vascular and/or biliary involvement of 1 lobe, no regional involvement, no metastasis). We adopted a conservative management approach because removing the lesions would require major surgery and because the results of Em18 serologic testing and ^18^F-FDG-PET scan suggested low-viability parasites (*1*,*13*). At the time of publication, the patient was receiving albendazole (400 mg 2×/d) with fat-containing meals and tolerating the medication well. Follow-up with contrast-enhanced ultrasound and serologic testing was scheduled every 6 months, MRI in 1 year, and ^18^F-FDG-PET scan in 2 years (*1*).

## Conclusions

AE is a complex disease with a high fatality rate (0%–25% 10-year survival) if untreated (*1*). It primarily affects the liver and is characterized by infiltrating, metastatic, tumor-like behavior (*1*). Unlike CE, AE lesions have no pathognomonic signs on imaging, and the differential diagnosis is mainly tumors (*1*). Even in AE-endemic areas, misdiagnosis and consequent incorrect treatment occurs frequently (*1*; Appendix reference *17*).

Curative treatment options include surgery and albendazole if radical resection is achievable, or albendazole alone indefinitely in other cases (*1*). Treatment interruption can be envisaged in selected cases when serologic testing and ^18^F-FDG-PET scans become negative (*1*). In this case, the Western blot banding pattern, low antibody concentrations against crude parasite antigens, negativity of Em18 ELISA, and faint hypermetabolism on ^18^F-FDG-PET scan indicate low parasite viability (*1*,*13*). PCR on bioptic material was negative, explained by the absence of cell-containing larval structures on histology; however, *E. multilocularis*–specific immunochemistry was positive, confirming the diagnosis (*1*,*13*).

A report from 1928 mentioned 2 human AE cases identified in South Tyrol in 1906 and 1922 (*2*), but reports of human confirmed AE in Italy are otherwise lacking; a 2019 research review identified no reports from this country (*12*). Italian Hospital Discharge Records reports cases labeled as AE according to International Classification of Diseases, 9th Edition (Appendix reference *18*). From the analysis of cases that we could examine (Appendix reference *19*), those cases seem to be CE with multiloculated cyst morphology (CE2 and CE3b stages according to WHO-IWGE), erroneously recorded as *E. multilocularis* (*1*).

An expanding area of endemicity of *E*. *multilocularis* in Europe has been observed and predicted by modeling (*3*). Because of the high lethality of this disease if misdiagnosed and mistreated, physicians, especially in Italy’s alpine regions, must be informed about this infection and its possibility even in patients who have never lived in or traveled to known endemic areas.

Appendix Additional information about a confirmed autochthonous case of human alveolar echinococcosis, Italy, 2023
