# Supplementary material for: Confirmed Autochthonous Case of Human Alveolar Echinococcosis, Italy, 2023
Source: Emerg Infect Dis. 2024 Feb;30(2):350–3. doi: 10.3201/eid3002.231527 (PMC10826761; doi:10.3201/eid3002.231527)
Supplement: Appendix — Additional information about a confirmed autochthonous case of human alveolar echinococcosis, Italy, 2023 [file 23-1527-Techapp-s1.pdf]

Article DOI: https://doi.org/10.3201/eid3002.231527

EID cannot ensure accessibility for supplementary materials supplied by authors. Readers who have difficulty accessing supplementary content should contact the authors for assistance.

Confirmed Autochthonous Case of Human Alveolar Echinococcosis, Italy, 2023

Appendix

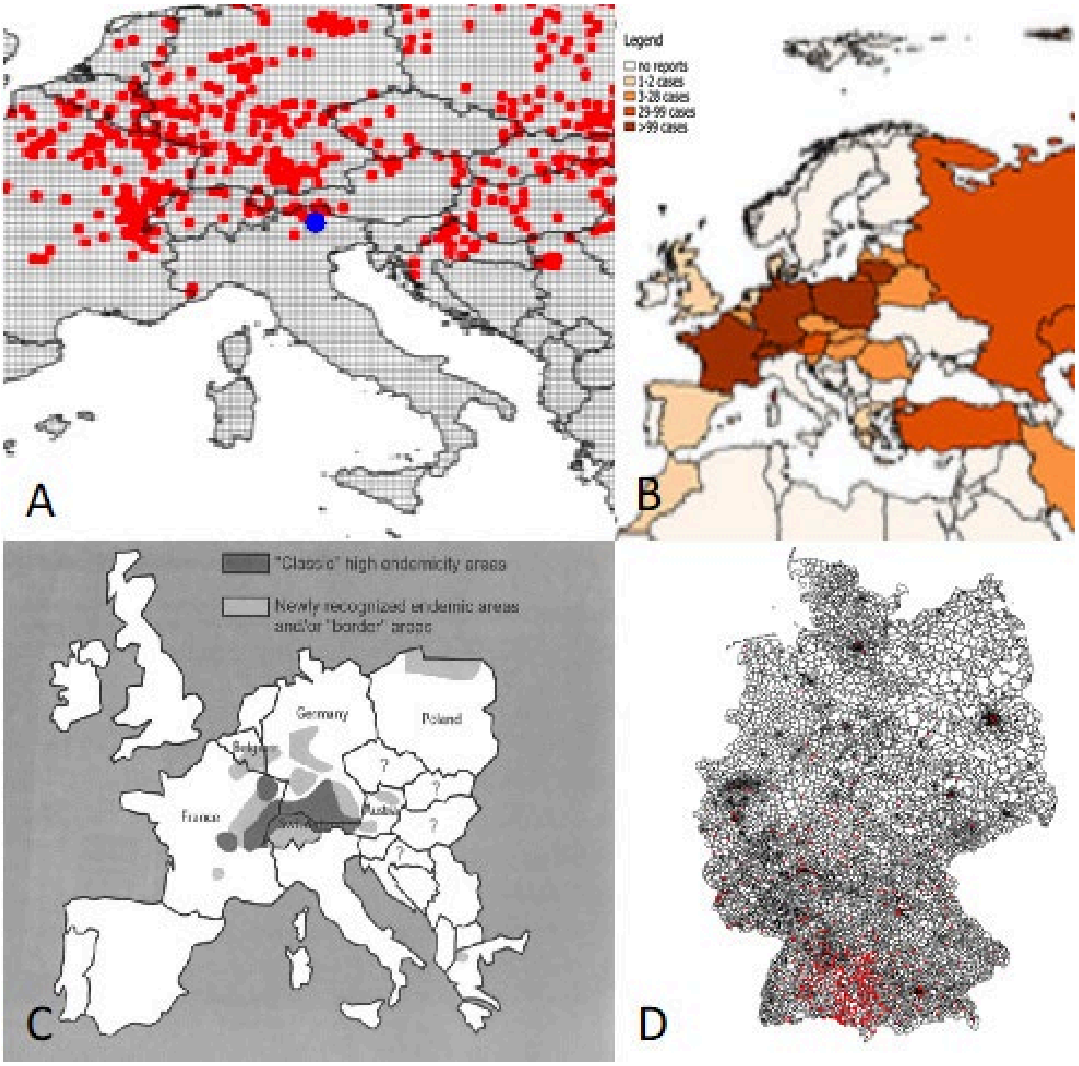

Appendix Figure. Maps of the distribution of *E. multilocularis* in Italy and adjacent countries. (A) *E. multilocularis* infection in wild carnivores (red dots) and living place of our human AE case (blue dot);

modified from reference *3* in the main text. (B) Distribution of AE in humans in central Europe according to the published literature 2001–2018; modified from reference *11* in the main text. (C) Endemic areas for human alveolar echinococcosis in Europe according to data from the European registry of alveolar echinococcosis (EurEchinoReg); from (*20*). (D) Distribution of AE cases in Germany based on data from the German database for AE in 2016; from (*21*).

Additional References

16. Stijnis K, Dijkmans AC, Bart A, Brosens LA, Muntau B, Schoen C, et al. *Echinococcus vogeli* in immigrant from Suriname to the Netherlands. Emerg Infect Dis. 2015;21:528–30. PubMed https://doi.org/10.3201/eid2103.141205

17. Stojkovic M, Mickan C, Weber TF, Junghanss T. Pitfalls in diagnosis and treatment of alveolar echinococcosis: a sentinel case series. BMJ Open Gastroenterol. 2015;2:e000036. PubMed https://doi.org/10.1136/bmjgast-2015-000036

18. Crotti S, Brustenga L, Cruciani D, Bonelli P, D'Avino N, Felici A, et al. Molecular screening of *Echinococcus* spp. and other cestodes in wild carnivores from Central Italy. Vet Sci. 2023;10:318. PubMed https://doi.org/10.3390/vetsci10050318

19. Cassini R, Canali M, Tamarozzi F, Angheben A, Capelli G, Gobbi F, et al. A One-Health evaluation of the burden of cystic echinococcosis and its prevention costs: case study from a hypo-endemic area in Italy. One Health. 2021;13:100320. PubMed https://doi.org/10.1016/j.onehlt.2021.100320

20. Vuitton DA, Zhou H, Bresson-Hadni S, Wang Q, Piarroux M, Raoul F, et al. Epidemiology of alveolar echinococcosis with particular reference to China and Europe. Parasitology. 2003;127(Suppl):S87–107. PubMed https://doi.org/10.1017/S0031182003004153

21. Schmidberger J, Kratzer W, Stark K, Grüner B; Echinococcosis Working Group. Alveolar echinococcosis in Germany, 1992-2016. An update based on the newly established national AE database. Infection. 2018;46:197–206. PubMed https://doi.org/10.1007/s15010-017-1094-0
